# Curriculum Development of a Clinical Course on Age-Friendly Health System 4Ms Care in Nursing Homes

**DOI:** 10.1093/geroni/igaf122.3911

**Published:** 2025-12-31

**Authors:** Megan Kazakoff, Yurun Cai, Beth Schwartz, Jacqueline Dunbar-Jacob, Elizabeth Schlenk

**Affiliations:** University of Pittsburgh, Pittsburgh, Pennsylvania, United States; University of Pittsburgh, Pittsburgh, Pennsylvania, United States; University of Pittsburgh, Pittsburgh, Pennsylvania, United States; University of Pittsburgh, Pittsburgh, Pennsylvania, United States

## Abstract

As the U.S. population ages, there is an urgent need to prepare nurses specialized in geriatric care, particularly in long-term care. Clinical curricula in nursing homes (NHs) are needed to enhance geriatric training for prelicensure students to improve patient outcomes. The purpose of this project was to design a practicum course with input from both NH staff and experienced instructors focused on principals of geriatric nursing care. University of Pittsburgh School of Nursing developed and implemented an 8-week, 1-credit elective clinical course based on the Age-Friendly Health Systems (AFHS) 4Ms framework through an academic-practice partnership with The Willows NH. Undergraduate nursing students engaged in weekly learning activities and developed a case study applying the 4Ms. Evaluation included pre- and post-course surveys of student and faculty competency ratings. Post-course data showed positive trends in student competency, especially in clinical assessment skills and person-centered care. Qualitative feedback highlighted improved understanding of geriatric care and nurse-resident relationships. The AFHS 4Ms-based course demonstrates that NHs can be effective clinical learning environments. Structured and competency-based curricula can enhance student preparedness for geriatric nursing. By leveraging structured orientation, strong academic-practice collaboration, and reflective learning, nursing programs can prepare students to meet the complex needs of aging populations in a way that also challenges misperceptions about NH practice.

